# Early Pro-Inflammatory Signal and T-Cell Activation Associate With Vaccine-Induced Anti-Vaccinia Protective Neutralizing Antibodies

**DOI:** 10.3389/fimmu.2021.737487

**Published:** 2021-10-11

**Authors:** Jue Hou, Shuhui Wang, Dan Li, Lindsay N. Carpp, Tong Zhang, Ying Liu, Manxue Jia, Hong Peng, Chang Liu, Hao Wu, Yunda Huang, Yiming Shao

**Affiliations:** ^1^State Key Laboratory of Infectious Disease Prevention and Control, National Center for AIDS/STD Control and Prevention, Chinese Center for Disease Control and Prevention, Beijing, China; ^2^Vaccine and Infectious Disease Division, Fred Hutchinson Cancer Research Center, Seattle, WA, United States; ^3^Center for Infectious Diseases, Beijing You’an Hospital, Capital Medical University, Beijing, China

**Keywords:** systems immunology, vaccinia vaccination, neutralizing antibodies, T-cell activation, inflammation

## Abstract

Both vaccine “take” and neutralizing antibody (nAb) titer are historical correlates for vaccine-induced protection from smallpox. We analyzed a subset of samples from a phase 2a trial of three DNA/HIV-1 primes and a recombinant Tiantan vaccinia virus-vectored (rTV)/HIV-1 booster and found that a proportion of participants showed no anti-vaccinia nAb response to the rTV/HIV-1 booster, despite successful vaccine “take.” Using a rich transcriptomic and vaccinia-specific immunological dataset with fine kinetic sampling, we investigated the molecular mechanisms underlying nAb response. Blood transcription module analysis revealed the downregulation of the activator protein 1 (AP-1) pathway in responders, but not in non-responders, and the upregulation of T-cell activation in responders. Furthermore, transcriptional factor network reconstruction revealed the upregulation of AP-1 core genes at hour 4 and day 1 post-rTV/HIV-1 vaccination, followed by a downregulation from day 3 until day 28 in responders. In contrast, AP-1 core and pro-inflammatory genes were upregulated on day 7 in non-responders. We speculate that persistent pro-inflammatory signaling early post-rTV/HIV-1 vaccination inhibits the nAb response.

## Highlights

After vaccinia virus helped eradicate smallpox, routine inoculation of the general public ceased. Currently, the vaccinia virus is being investigated as a vector for viral and tumor vaccines. While neutralizing antibodies (nAbs) are an established correlate of protection, not all vaccine recipients develop a protective nAb response. Considering the potential bioterrorist threat of smallpox and an increasingly unvaccinated population, it is imperative to understand the mechanisms driving the development of a protective nAb response. System immunology offers an opportunity to understand and compare the molecular signatures of sero-responders *versus* those of non-responders.

## Introduction

The global eradication of smallpox was a monumental accomplishment in public health that was achieved through vaccination with the vaccinia virus, with 11 strains used worldwide as part of this campaign (1). The immune response to multiple strains (e.g., Dryvax, Lister, and NYCBH) has been extensively characterized: primary vaccinia virus-specific CD4^+^ and CD8^+^ T-cell responses peak 2 weeks after inoculation (2), neutralizing immunoglobulin M (IgM) antibodies are induced within days after inoculation (3), and a robust neutralizing immunoglobulin G (IgG) response is raised within 2–3 weeks (4–6), leading to seroconversion in vaccinia-naive and vaccinia-experienced participants (7–9). In China, millions of individuals were inoculated with the replication-competent Tiantan strain of vaccinia virus (TVV) as part of the smallpox eradication campaign. However, after smallpox was declared eradicated by the World Health Organization in 1980, routine TVV immunization of the general public in China was stopped. No large-scale clinical study has been conducted in China to systematically characterize TVV-induced immune responses using modern systems immunology approaches.

Considering concerns regarding emerging zoonotic poxviruses (10), the categorization of smallpox virus as a category A bioterrorism agent (11) and a global population increasingly susceptible to smallpox (due to the discontinuation of vaccination), it is imperative to understand the mechanisms driving the development of a protective response. Historically, two vaccine-induced immune responses have been considered to be correlates of protection against smallpox disease: 1) serum neutralizing antibody (nAb) titer >1:32 (12) and 2) the presence of vaccine “take” (i.e., formation of a pustule at the inoculation site) (13). Significant heterogeneity in the IgG nAb response has been observed across individuals vaccinated with the vaccinia virus (3), and some vaccinated individuals do not mount a nAb response (titer < 1:32) despite observable vaccine take (14). This finding suggests that the current understanding of poxvirus vaccine-mediated protection is incomplete.

The vaccinia virus genome is amenable to large inserts, and there is an increasingly reduced proportion of vaccinia-experienced individuals in the general population due to the discontinuation of vaccination against smallpox. Thus, recombinant vaccinia viruses are considered to have excellent potential as vaccine vectors (15, 16). Currently, the replication-competent TVV is being developed as an HIV-1 vaccine vector in China, the recombinant Tiantan vaccinia-based HIV-1 vaccine (rTV/HIV-1). rTV/HIV-1 has been shown to provide complete protection in Chinese rhesus macaques from intravenous homologous simian HIV challenge (17) and was advanced to a double-blind, placebo-controlled phase 1 trial conducted in China (18), which consisted of a regimen of three DNA/HIV-1 vaccine primes, followed by a single rTV/HIV-1 booster. A phase 2a trial was next conducted by the China Centers for Disease Control (ClinicalTrials.gov identifier: NCT01705223). This phase 2a trial provided a unique opportunity to study the response to the Tiantan vaccinia virus, in the context of the rTV/HIV-1 booster. Inspired by previous systems vaccinology studies (19–29), we designed a sub-study focused on understanding the kinetics of the rTV/HIV-1 booster vaccination-induced vaccinia-specific immune activation, determining whether and how the transcriptomic profiles correlate with the immune responses and investigating the transcriptomic signatures of responders (Rs) and non-responders (NRs).

## Materials and Methods

### Study Cohort

The phase 2a trial (ClinicalTrials.gov identifier: NCT01705223) enrolled a total of 150 HIV-uninfected healthy men and women to evaluate the safety and immunogenicity of a HIV-1 clade B′/C DNA vaccine (DNA/HIV-1) followed by an rTV/HIV-1 booster. Trial participants received three DNA/HIV-1 primes at weeks 0, 4, and 8, followed by a single subcutaneous dose of rTV/HIV-1 (Beijing Biological Preparations Institute Co., Ltd., Beijing, China). For the vaccinia sub-study, specimens from 15 participants who received the entire vaccination series were used. The Ethics Committee of the Chinese CDC approved this phase 2a trial (Ethics Committee Project Identifier: X111012202). All participants signed a written informed consent form prior to the initiation of the study procedures.

### Study Products

The DNA/HIV-1 vaccine consisted of two plasmids encoding the gp140 and the *gag/pol/nef* genes of clade B′ HIV-1 and clade C HIV-1, while the rTV/HIV-1 vector encoded the *gag*, *pol*, and *gp140* genes of the circulating HIV-1 recombinant form CRF/07_BC/CN54 (17).

### Sample Collection

**Figure 1:**
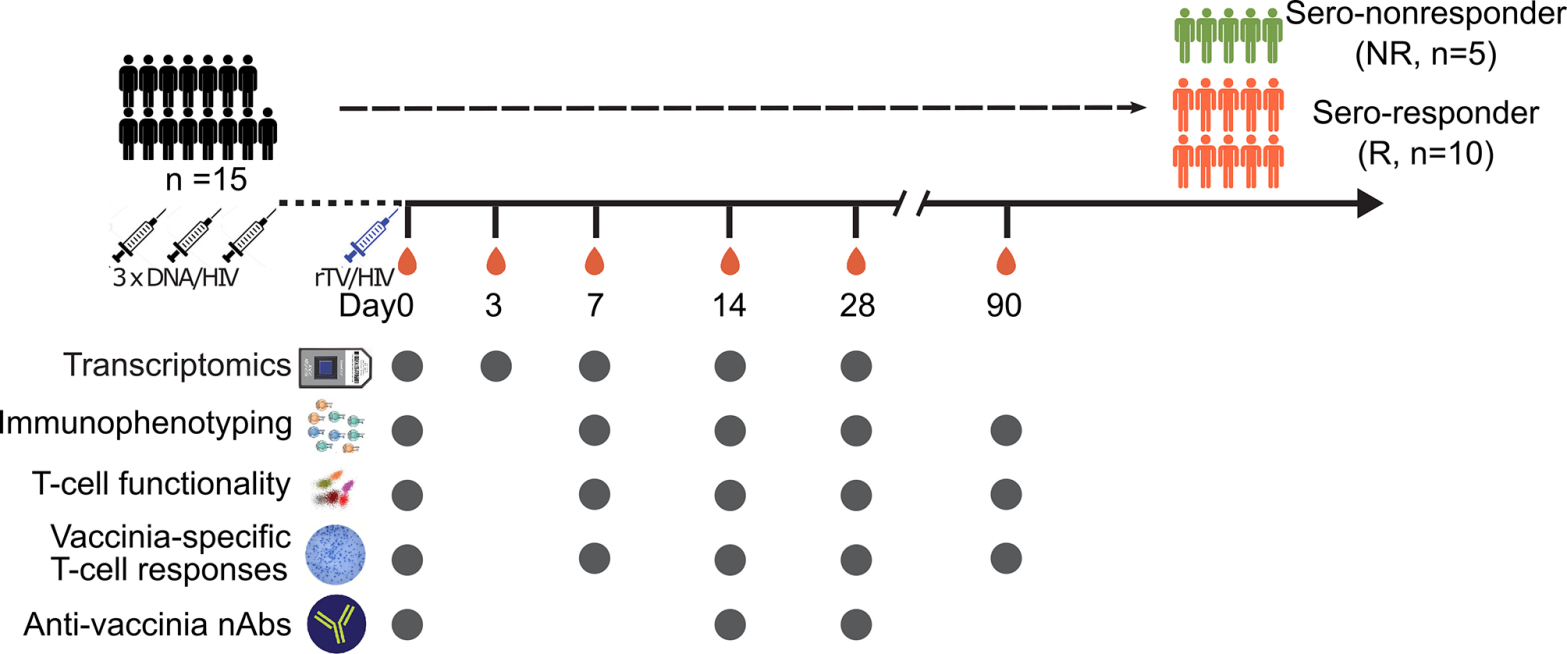
Vaccination schedule and sampling scheme. Schematic diagram of the vaccinations and sample collection. Fifteen subjects participated in this sub-study. All participants were given three DNA/HIV-1 vaccine primes at weeks 0, 4, and 8, followed by a recombinant Tiantan vaccinia-based HIV-1 vaccine (rTV/HIV-1) booster, at 6 months post-last DNA/HIV-1 vaccine prime. Peripheral blood mononuclear cells and serum samples were taken on day 0 prior to the rTV/HIV-1 immunization and then again on days 3, 7, 14, 28, and 90 post-rTV/HIV-1 booster vaccination. *Filled circles* indicate the data collection time points.

Blood was drawn from each volunteer into EDTA-2K Vacutainer tubes (BD Biosciences, Franklin Lakes, NJ, USA) on the day of the rTV/HIV-1 boost (day 0, pre-vaccination) and on days 3, 7, 14, 28, and 90 thereafter. Peripheral blood mononuclear cells (PBMCs) were isolated from these samples using standard Ficoll-Paque Plus (GE Healthcare, Chicago, IL, USA) density gradient centrifugation and frozen in dimethyl sulfoxide with 10% fetal bovine serum (FBS) in liquid nitrogen. The assays performed at the different sample collection time points are shown in **Figure 1**.

### Quantitative Firefly Luciferase-Based Vaccinia Virus-Neutralizing Antibody Assay

Plasma samples were heated to 56°C for 60 min to inactivate complement and serially diluted with Dulbecco’s modified Eagles’ medium (DMEM) supplemented with 2% FBS. Diluted serum (100 µl) was distributed into each of three corresponding wells in a 96-well plate, 50 µl of the rTV-Fluc virus was added to each well, and the plates were incubated at 37°C/5% CO_2_ for 1 h. Following incubation, 100 µl of Vero cell suspension was added to each well. The plates were placed on a shaker for 1 min and incubated at 37°C/5% CO_2_ for up to 48 h. After incubation, 100 µl of the supernatant was aspirated and 100 µl of D-luciferin substrate (Caliper, Waltham, MA, USA) was added into each well. The plate was incubated in the dark at room temperature for 2 min, after which luminescence was measured using a GLOMAX 96 microplate luminometer (Promega, Madison, WI, USA). The 50% neutralization titer (NT_50_) for each serum sample was defined as the serum dilution at which the relative light unit (RLU) was reduced by 50% compared with the virus-containing control wells after subtraction of the background RLU in the cell-containing control wells. Titers >30 (the assay lower limit of detection) were considered positive (30). Anti-vaccinia nAbs were measured by the firefly luciferase-based assay as described previously (30). “Responders” (*n* = 10) were defined as participants whose nAb titer was >1:30 in at least three time points post-boost, and “non-responders” (*n* = 5) were defined as participants whose nAb titer did not exceed 1:30 in at least three time points post-boost.

### Flow Cytometry and ELISpot

Complete blood counts were conducted on fresh blood samples to obtain cell counts for each blood cell type and hemoglobin concentrations. For cell phenotyping, 1 million PBMCs from each sample were surface stained with the antibody cocktails shown in **Supplementary Table S1** and with Live/Dead staining reagents (Life Technologies, Carlsbad, CA, USA) to assess cell viability. Staining was performed at 4°C in the dark for 30 min. For the immunostaining of intracellular proteins, cells were fixed and permeabilized with Fix/Perm (eBioscience, San Diego, CA, USA) for 30 min at 4°C in the dark prior to antibody incubation.

For analysis of natural killer (NK) cell-mediated antibody-dependent cellular cytotoxicity (ADCC), an established assay was used (31). In this assay, the target cells were coated with anti-P815 antibodies to serve as the Fc target cells for NK cell-mediated ADCC. The cells were then co-cultured with PBMCs as effector cells and NK cell function was assessed. Briefly, 0.2 million K562 cells (human chronic myelogenous leukemia cells, maintained in our laboratory) were cultured with P815-specific antibodies (Accurate Chemical & Scientific, Carle Place, NY, USA) for 1 h at 37°C in 5% CO_2_. Antibody-coated P815 cells were washed twice with ice-cold RPMI medium containing 10% FBS and used as the target cells. The target cells were co-cultured with 1 million PBMCs (effector cells) and then incubated with anti-CD107a antibodies and Golgi-Stop (BD Biosciences) for 5 h at 37°C in 5% CO_2_. Following culture, the samples were processed according to standard protocols and stained intracellularly with Alexa Fluor 700-conjugated anti-interferon gamma (IFNγ) and fluorescein isothiocyanate (FITC)-conjugated anti-tumor necrosis factor alpha (TNFα) antibodies.

For the quantification of T-cell function, 1 million PBMCs were infected with the parent rTV vector (no HIV-1 inserts) at a multiplicity of infection (MOI) of 1 or stimulated with phorbol myristate acetate (PMA) as a positive control. Brefeldin A (BFA) was added to each well, after which all the samples were incubated for 6 h at 37°C in 5% CO_2_. The cells were permeabilized and stained intracellularly using phycoerythrin (PE)-conjugated anti-interleukin 2 (IL-2), Alexa Fluor 700-conjugated anti-IFNγ, and allophycocyanin (APC)-conjugated anti-TNFα antibodies. Events were acquired on a BD LSRFortessa instrument (BD Biosciences) and analyzed using FlowJo software (TreeStar, Ashland, OR, USA). The antibodies used for immunostaining are listed in **Supplementary Table S1**.

Vaccinia-specific IFNγ-secreting T cells were detected by enzyme-linked immunospot (ELISpot) assay. Plates were coated with purified anti-human IFNγ at a concentration of 5 μg/ml, incubated at 4°C overnight, and washed and blocked for 2 h at room temperature. PBMCs (2 × 10^5^) were added to the wells in duplicate. The cells were stimulated with vaccinia virus at a MOI of 1. The positive control was stimulated with PMA at 50 ng/ml and ionomycin at 1 μg/ml; the negative control was stimulated with medium alone. The PBMCs were incubated at 37°C and 5% CO_2_ for 24 h and then lysed with sterile water. The plates were washed three times with PBST (phosphate-buffered saline with Tween 20) prior to a 1-h incubation with biotinylated anti-human IFNγ antibody, followed by the addition of streptavidin–horseradish peroxidase (HRP) at 37°C for 1 h. The plates were washed again and developed with 100 µl of a 3-amino-9-ethylcarbazole (AEC) substrate solution for approximately 30 min. The reaction was stopped by washing the plate with distilled water. IFNγ spots were analyzed by an automated ELISpot plate reader (ImmunoSpot, Cleveland, OH, USA). Spot-forming cells (SFCs) were quantitated as the average number of spots in duplicate wells per 10^6^ PBMCs.

### RNA Preparation and Microarray

After isolation, 1 × 10^6^ PBMCs were immediately lysed in 1 ml of TRIzol (Life Technologies) and stored at −80°C until sample processing. For processing, the samples were thawed and the RNA extracted following the manufacturer’s instructions. The quality of the RNA was assessed using a Nanodrop 2000 Spectrometer (Thermo Scientific, Waltham, MA, USA) and by visualizing the integrity of the 28S and 18S bands on an Agilent Bioanalyzer 2100 instrument (Agilent Technologies, Santa Clara, CA, USA).

Qualified total RNA was further purified using a RNeasy Micro Kit (QIAGEN, Hilden, Germany). Contaminating genomic DNA was removed using an RNase-Free DNase Set (QIAGEN). The purified RNA was stored at −80°C until microarray analysis.

To obtain biotin-labeled complementary RNA (cRNA), total RNA was amplified, labeled, and purified using the GeneChip 3′IVT Express Kit (Affymetrix, Santa Clara, CA, USA) following the manufacturer’s instructions. After hybridization on Human PrimeView Arrays for 16 h at 45°C and rotation at 60 Work4Perpm in a Hybridization Oven 640 (Affymetrix), the slides were washed and stained with a Fluidics Station 450 system (Affymetrix). Scanning was performed on a seventh-generation GeneChip Scanner 3000 (Affymetrix). Affymetrix GCOS software was used to perform image analysis and generate raw intensity data.

All microarray data analysis was performed in R (version 3.5.2) (32). Initially, data quality was assessed by determining the background level, 3′ labeling bias, RNA quality, and pairwise correlation among the samples. For the PrimeView chip, the customized CDF file (version 22, ENTREZG) downloaded from the BrainArray website was performed in probe set mapping. Normalization was performed with the RMA algorithm, which includes global background adjustment and quantile normalization. Interquartile range (IQR) was applied for raw data filtering using the genefilter package (version 1.64.0) (33), and the threshold was set to remove genes with an IQR less than the 50th percentile of the IQRs across all genes. The sample gene intensities were log_2_ transformed in all subsequent analyses. Genes were annotated using information from NCBI (34) (modified April 25, 2017). These data have been deposited in the NCBI Gene Expression Omnibus and are accessible through GEO Series accession number GSE118976.

### Bioinformatics Analyses

#### Statistical Calculation on Immunological Data

Immune response data were plotted using the ggstatsplot (version 0.3.1) (35) package in R. Bar graphs display the mean values with standard deviation. One-way analysis of variance (ANOVA) with Dunnett’s multiple comparison procedure was used to compare the immune responses at multiple time points post-rTV vaccination *vs.* their baseline values. The Wilcoxon signed-rank test was used to compare the nAb data between different time points. Pearson’s correlation coefficients were calculated and the data were visualized using the corrplot package (version 0.84) (36) in R. Statistical significance was declared when the resulting *p*-value was less than 0.05.

#### Identification of Differentially Expressed Genes and Blood Transcription Modules After rTV/HIV-1 Vaccination

The limma package (version 3.38.3) (37) was used to identify the differentially expressed genes (DEGs) and the blood transcriptional modules (BTMs). DEGs on a given day post-boost were defined as genes whose average expressions on that day across all 15 participants had a Benjamini–Hochberg (BH)-adjusted *q*-value less than 0.05. Correlations between measurements and the array quality weights were estimated and then input into the linear model fit. Empirical Bayes moderation of the standard error and the BH false discovery rate (FDR) correction for multiple testing were employed.

BTM analysis (27) was performed by summarizing the pre-processed (filtered and normalized) expression values into module-level enrichment scores *via* the Gene Set Variation Analysis R package (version 1.30.0) (38). The analysis was performed within Rs and within NRs. BTMs with significantly decreased or increased scores at each time point were defined as those having a BH-adjusted *q*-value less than 0.05.

Gene set enrichment analysis (GSEA) was performed on the pre-ranked gene list using BTMs as the gene sets. Gene expressions on days 14 and 28 were ranked based on the nAb responses on days 14 and 28, respectively. GSEA was then run in the pre-ranked list mode with 1,000 permutations to generate normalized enrichment scores for the BTMs based on the distribution of the member genes of each module in the ranked list. The list of pre-ranked genes and BTM associations was generated using Circos 0.66 (http://circos.ca).

#### Weighted Gene Co-Expression Network Analysis

To identify the gene module signatures of the Rs and NRs, the R package WGCNA (version 1.69) (39) was used. Briefly, to construct the weighted signed co-expression network, genes with an IQR less than the 50th percentile of the IQRs across all genes were selected, yielding a total of 9,317 genes. To examine the correlations of the gene modules with cellular function, phenotype, and nAb titer, microarray data and immune responses from day 0, 7, 14, and 28 were selected for the construction of the co-expression network. To this end, the consensus network (Rs and NRs) and the individual networks for Rs and NRs were constructed separately. We quantified the associations of the individual gene expression levels with the measured cellular and humoral immune responses to vaccination, defined as gene significance (GS) correlations. For each module, we also calculated a quantitative measure of module membership (MM), defined as the correlation between the module eigengene and the gene expression profile. The MM enables quantification of the correlation significance of each immune response with the potentially most correlated module. For selected modules with strong positive (magenta, turquoise, and red modules) or negative (brown and cyan modules) correlations with the vaccinia nAb titers, we filtered the genes (GS > 0.6 and MM > 0.6) to present and visualize the relationship between highlight genes and nAb using Cytoscape (40). The functionality of these top genes was analyzed using the clusterProfiler (version 3.10.1) (41) for BTM enrichment.

#### Reconstruction of Transcriptional Networks and Analysis of Master Regulators

To understand which critical transcription factors (TFs) participate in vaccine-induced immune responses and how they contribute to the generation of a nAb response, transcriptional regulatory networks were reconstructed and analyzed for regulons using mutual information of the gene expression data using the Reconstruction of Transcriptional regulatory Networks (RTN) package (version 2.6.3) (42, 43) in R. A TF database (44) was imported and 795 TFs present in the processed expression data were used as input seeds for transcriptional network inference. Transcriptional network analysis was performed at each time point after converting from the pre-processed inferred transcriptional networks. Two-tailed GSEA tests were used for enrichment analysis of the transcriptional networks. The results were summarized and visualized using the ComplexHeatmap package (version 1.20.0) (45). TFs identified at each time point were mapped to the Human Protein Reference Database (HPRD) to construct a protein–protein interaction network using RedeR (version 1.30.0) (46). The network was visualized in Cytoscape (40).

#### Inflammation Index and Pseudo-Infection Index Calculation

Twenty-seven inflammatory signature genes have been previously identified (47) that participate in a positive feedback loop among the IL1/NF-kb pathway (*IL1A*, *IL1B*, *IL1R1*, *IL1R2*, *IL1RAP*, *IL1RL1*, *MYD88*, *IRAK2*, *NFKB1* and *NFKB2*), the IL6/STAT3 pathway (*IL6*, *LIF*, *OSMR*, *JAK2*, and *STAT3*), the TNF/activator protein 1 (AP-1) pathway (*TNFSF10*, *TNFRSF10D*, *TNFRSF11B*, *TNFRSF21*, *ATF3*, *FOS*, *FOSL1*, *FOSL2*, *JUN*, and *JUNB*), and MAP kinases (*MAP3K8* and *MAP4K4*).

The inflammatory index was calculated based on the log2 expression levels of these genes (*E_ij_* refers to gene *i* in sample *j*), as reported previously (47). The total gene number to calculate the index is *n* (*n* ≤ 27) and the total sample number is *m*. The gene expression intensity was normalized across all samples to their median values, calculated as *N_ij_* = *E_ij_* − median (*E_i_*_1_, *E_i_*_2_, … *E_im_*). For each sample *j*, the median expression intensity of the signature genes was calculated as *S_j_* = median (*N*_1_*_j_*, *N*_2_*_j_*, …. *N_nj_*). The lower 5 percentile of the expression intensity for each gene across samples was set as the baseline of inflammatory levels (*B_i_*) and the median expression level calculated as *M_i_*. The final inflammatory index for a sample *j* was calculated as *S_j_* + median ((*M*_1_ − *B*_1_), (*M*_2_ − *B*_2_),…, (*M_n_* − *B_n_*)).

## Results

### Study Design and Participants

For this analysis, the samples used were from a subset of participants (*n* = 15, age range = 26–51 years, mean ± SD = 38.46 ± 8.04) in the phase 2a DNA/HIV-1 prime, rTV/HIV-1 booster trial. The study design is illustrated in **Figure 1**.

### Correlation of rTV/HIV-1-Induced Cellular and Humoral Responses

**Figure 2:**
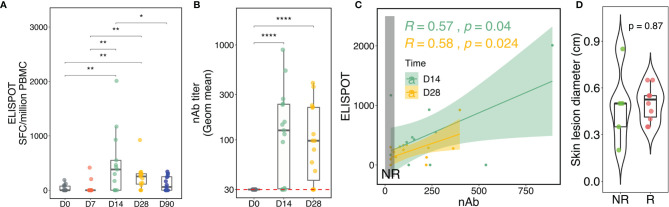
Kinetics of the vaccinia-specific T-cell response, neutralizing antibody (nAb) titer, and skin lesion after the recombinant Tiantan vaccinia-based HIV-1 vaccine (rTV/HIV-1) booster vaccination. **(A)** Vaccinia virus-specific T-cell responses as assessed by enzyme-linked immunospot (ELISpot). Individual values are presented in *box plots*. *SFCs*, spot-forming cells. **(B)** Kinetics of the nAb response to the vaccinia virus. **(C)** Correlations between the vaccinia-specific ELISpot responses and the nAb titers on days 14 and 28. *Light green* and *light mustard shading* indicate the 95% confidence intervals of the two trend lines. The *vertical dark grey rectangle* encloses responses for non-responders (NRs). **(D)** Skin lesion diameter at the inoculation site as measured 1 week after rTV/HIV-1 booster vaccination. For all, *p*-values for significant difference between two given time points were calculated using the Wilcoxon test. *P < 0.05, **P < 0.01, ****P < 0.0001.

We began by assessing the kinetics of the vaccinia-specific cellular and humoral responses to rTV/HIV-1 vaccination. rTV/HIV-1 vaccination induced high vaccinia-specific IFNγ T-cell responses on day 14; these responses declined over time, but remained detectable through day 90 (**Figure 2A**). The vaccinia-specific CD8^+^ T-cell responses waned more rapidly than did the vaccinia-specific CD4^+^ T-cell responses on days 28 and 90 (**Supplementary Figures S1A, B**). Moreover, the vaccination also elicited the vaccinia-specific IFNγ NK cell responses on day 14 (**Supplementary Figure S1C**). Anti-vaccinia nAbs were detectable on day 14 and were maintained at a similar level through day 28 (**Figure 2B**). As substantial variability was observed across participants’ vaccinia-specific T-cell responses and nAb responses, we investigated correlations at the individual level. Moderate positive correlations between the nAb and T-cell responses were observed on days 14 and 28 (**Figure 2C**).

In some participants, nAb responses were not detected, enabling their characterization into Rs and NRs according to the vaccine-induced nAb titer (see *Materials and Methods*). In order to ascertain that nAb responsiveness was not led by failures of inoculation, vaccine “take” acted as an alternative measure to evaluate whether the vaccine established infection successfully in Rs and NRs because vaccinia vaccination induces barely detectable viremia (48, 49). Vaccine “take,” corresponding to the development of a lesion at the inoculation site, provides evidence of local viral replication and is generally held to be a correlate of vaccinia-specific immunity and clinical protection against smallpox (50). It was thus surprising that Rs and NRs alike all developed vaccine takes, with no significant difference in skin lesion size between the two groups (**Figure 2D**). This finding suggested that the vaccinia virus established infection similarly in Rs *versus* NRs and prompted us to further investigate the reasons for NRs exhibiting vaccine take, yet failing to mount a nAb response.

### Broad and Persistent Transcriptional Response in Responders to Vaccinia Vaccination, Characterized by T-Cell Activation and Suppression of Inflammation

We first employed the weighted gene co-expression network analysis (WGCNA) algorithm, which defines transcriptional modules based on gene co-expression and identifies gene clusters associated with particular immune response readouts, to examine the associations between the gene modules and vaccine-induced immune responses, including vaccinia-specific T-cell responses, anti-vaccinia nAbs, and the frequencies of different immune cell types circulating in the PBMC compartment. The dataset (9,317 gene transcripts) from Rs clustered into 11 distinct modules and identified “turquoise,” “red,” and “magenta” modules positively associated with CD8^+^ T cells and/or nAb titer, along with “brown” and “cyan” modules that were negatively correlated with nAb titer in Rs (**Supplementary Figure S2A**), and 12 differential modules were clustered in NRs, but no module was significantly associated with nAb response (**Supplementary Figure S2B**); therefore, subsequent analysis only focused on interpreting the Rs dataset.

**Figure 3:**
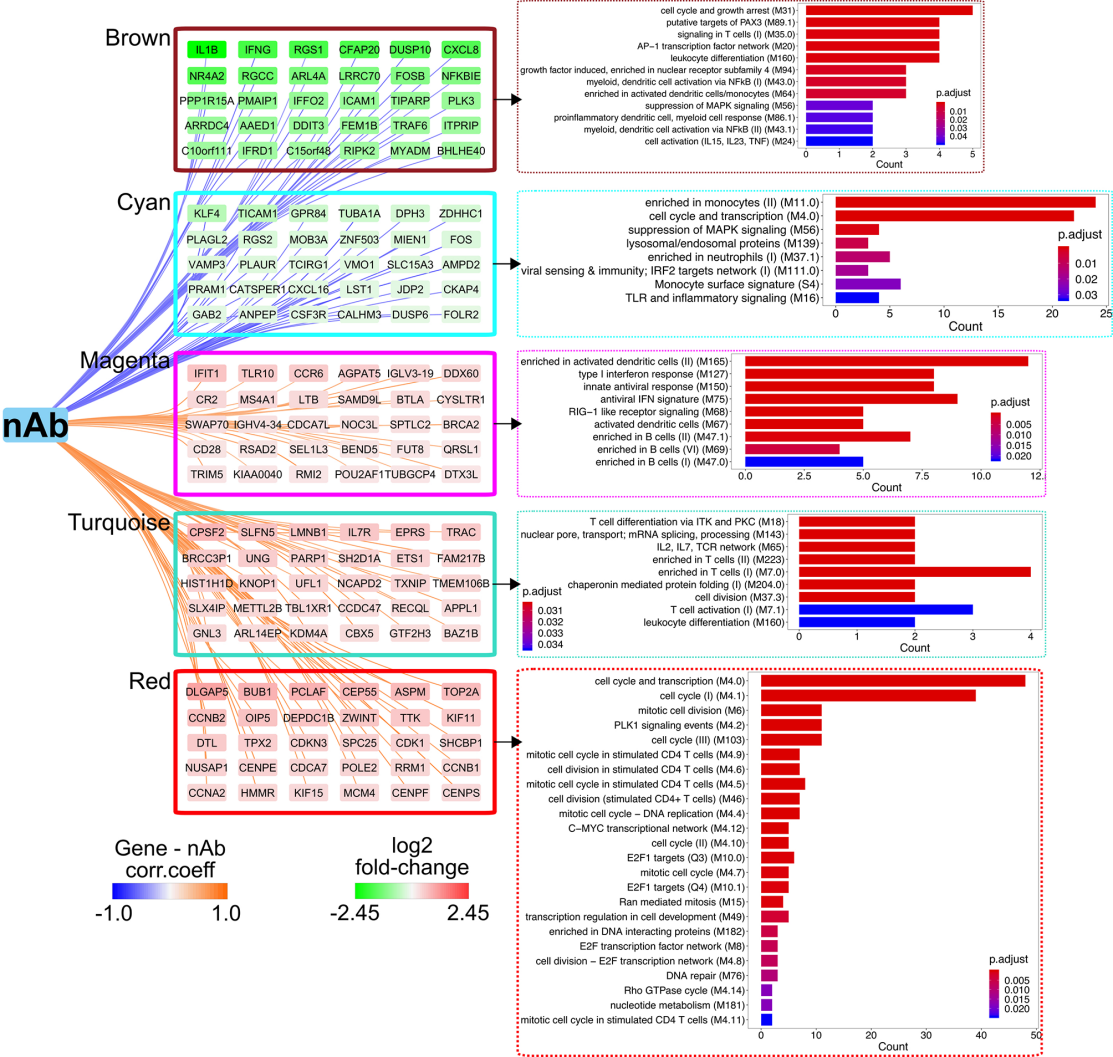
Weighted gene co-expression network analysis (WGCNA) identifies a co-expression network and critical genes associated with neutralizing antibody (nAb) production. Correlations of log_2_ fold change of individual genes in the brown, cyan, magenta, turquoise, or red modules with antibody (Ab) titer. *Edge width* denotes the correlation strength and *node color* represents the log_2_ fold change (day 14/day 0). Blood transcriptional modules (BTMs) enriched in each module as assessed by clusterProfiler are shown. *Bar plots* display the number of genes in each BTM, with genes ranked by the adjusted *p*-value for enrichment.

**Figure 4:**
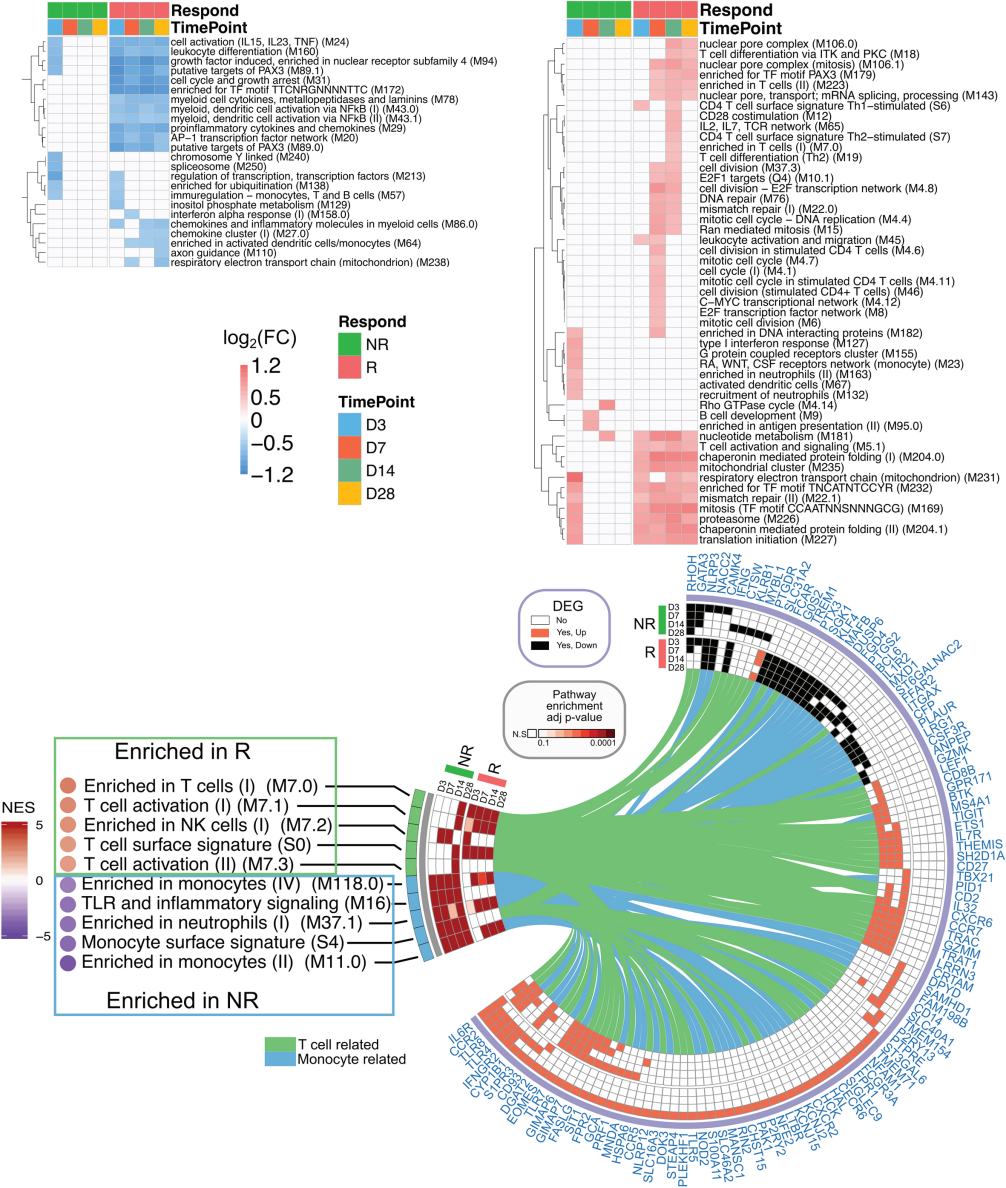
Transcriptional profiling of responders (Rs) and non-responders (NRs). **(A)** Heatmap showing blood transcriptional modules (BTMs) up/downregulated at least 1.2 fold change in the post-vaccination enrichment score compared to the baseline score and false discovery rate (FDR) < 0.05 after the recombinant Tiantan vaccinia-based HIV-1 vaccine (rTV/HIV-1) booster vaccination. *Green* (*left*), non-responders; *red* (*right*), responders. **(B)** Gene set enrichment analysis (GSEA) of the pre-ranked genes based on the neutralizing antibody (nAb) titers is shown in the *left panel*. *NES*, normalized enrichment score. Positive and negative enrichment denoted by *red* and *blue*, respectively. Circos plot represents the differentially expressed genes (DEGs) and the GSEA for the groups of NRs and Rs on day 3 through day 28. The BTM enrichment adjusted *p*-values are presented on the *left arc*, and the log_2_ fold change values compared to the baseline of DEGs belonging to each BTM are shown on *the right arc*. DEGs are connected to their corresponding BTM(s) with *lines*.

Overrepresentation analyses revealed that the top genes (in terms of strong gene–module relationships and/or high gene–immune response correlations; see *Materials and Methods*) in the brown, cyan, magenta, turquoise, and red modules were related to the AP-1 TF network, inflammation signatures, B cells, T-cell activation, and T-cell mitotic cycle, respectively (**Figure 3**). These findings imply that the AP-1 TF network and inflammation genetic signatures were suppressed in Rs, but T cells and B cells were active. Nevertheless, these co-expression networks were only constructed in Rs in order to characterize the landscape of transcriptional response in both Rs and NRs. The BTM-based gene sets were utilized to compare potential differences between the Rs and NRs and to identify the association of nAb and gene profiling (27). The NRs had almost no transcriptional response to rTV/HIV-1 vaccination, with the exception of a small day 3 response such as the activation of immune sensing modules and the downregulation of translation- and transcription-related modules (**Figure 4A**). In contrast, the Rs had a broad transcriptional response to rTV/HIV-1 vaccination, spanning the activation of modules related to cell cycle, metabolism, nuclear pore transport, translation, and T-cell activation/differentiation. Especially, the Rs showed persistent downregulation of multiple modules throughout the entire time course, including the “AP-1 transcription factor network (M20)” and “proinflammatory cytokines and chemokines (M29),” which was consistent with the results from the nAb titer metric pre-ranked GSEA on gene data on days 14 and 28 (**Figure 4B**). Through the pre-ranked GSEA, any gene set positively enriched can be considered to be positively associated with nAb titer, and *vice versa*. Consistent with the data presented in **Figure 4A**, the modules with the highest enrichment scores in the Rs were almost entirely related to T-cell activation (**Figure 4B**). In contrast, the modules with the largest negative enrichment scores in the NRs involved monocyte, neutrophil, and inflammation enrichment (**Figure 4B**). To visualize the individual genes that were present in modules with the largest positive and largest negative enrichment scores, we generated a Circos plot (**Figure 4B**). The small arc on the left shows the enrichment-adjusted *p*-values for the NRs and Rs at different time points, and the larger arc on the right shows whether the genes in each module were identified as DEGs. The links between the modules and genes indicate the relationships. The plot shows that the inflammation-related genes (*NALP3* and *PTX3*) and the innate sensing gene (*TREM1*) were downregulated in Rs, while genes related to T-cell status and differentiation (*CD8B*, *ETS1*, *CXCR6*, *CCR7*, *GZMM*, etc.) were upregulated.

The results suggest that nAb titers were positively associated with the T-cell response, but inversely correlated with inflammation.

### AP-1 Transcriptional Factor Complex Impacts on nAb Response

**Figure 5:**
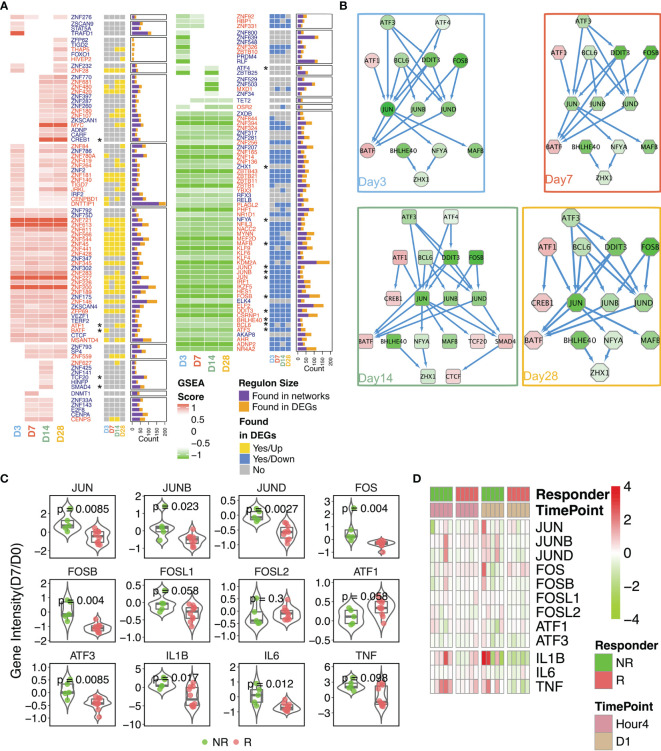
Reconstruction of the transcriptional networks and analysis of master regulators. Transcriptional networks were reconstructed with the expression data in responders. **(A)** The identified transcriptional regulatory units were found *via* network reconstruction. Gene names are shown in *red*. A *yellow square* indicates that the differentially expressed gene (DEG) was upregulated on the designated time point, a *blue square* indicates that the DEG was downregulated on the designated time point, and a *gray square* indicates that the DEG was not significantly differentially expressed on the designated time point. The size of each regulon manipulated by each transcription factor (TF) is given in the *stacked bar plot*. *Purple* indicates regulons found through the network analysis; *mustard* indicates regulons present as the DEGs. *Asterisks* denote genes involved in the interaction network . **(B)** Protein–protein interaction network generated from TFs within responders. The time point-specific network is shown in each *colored box*. Nodes are colored according to the log_2_ fold change (day 14 compared to day 0). **(C)** Comparison of the expression intensities of selected genes on day 7 (baseline-normalized) in non-responders (NRs) *versus* responders (Rs). *P*-values were calculated using the Wilcoxon test. Each *dot* in the *box*–*violin plot* represents a single participant. **(D)** Fold change in the gene expressions (compared to baseline) of the AP-1 pathway-related genes at hour 4 and day 1 post-inoculation.

Considering that the AP-1 TF network was suppressed over the entire time course in the Rs and was enriched in the brown module (which was inversely associated with nAb), we constructed transcriptional regulatory networks in Rs and NRs, with the aim of identifying master regulators (i.e., TFs) that potentially drive the development of a nAb response. Overall, 149 TFs were identified as differentially regulated in Rs (81 upregulated and 68 downregulated) (**Figure 5A**). The kinetics of TF changes varied across TFs. Some were persistently upregulated from day 3 through day 28, whereas other NRs exhibited transient upregulation, predominantly on day 3 (**Supplementary Figure S3**). As combinatorial interactions of multiple TFs can dictate the gene expression outcomes, a TF-coordinated interaction network was generated for each time point using the differentially expressed TFs in Rs. Across all time points (**Figure 5B**), AP-1 complex TFs (*ATF3*/*FOSB*/*JUN*) were downregulated and dominated the network. While these network hubs were downregulated, *ATF1* and *BATF* were persistently upregulated; *CREB1*, *TCF20*, *SMAD4*, and *CTCF* were highly expressed at later time points (days 14 and 28). However, the expression patterns of these TFs in Rs differed from those in NRs on day 7 (**Figure 5C**), in which AP-1 complex genes were highly upregulated in NRs, albeit with some degree of individual variability. Moreover, while the pro-inflammatory cytokines IL1β, IL6, and TNFα were significantly upregulated in NRs, they were all downregulated in Rs (**Figure 5C**). Aside from these later time points, we also found that AP-1 gene expressions were detected within 1 day post-rTV/HIV-1 vaccination (hour 4 and day 1), and both Rs and NRs showed a slight upregulation of *JUN*, *FOS*, and other AP-1 complex genes (**Figure 5D**), but with inter-individual variabilities.

These results imply that the AP-1 pathway was initiated in both Rs and NRs, but AP-1 pathway activation was persistent up to day 7 post-immunization in NRs, which demonstrated an aforementioned adverse correlation with nAb.

### Persistent Inflammation Associates With a Dampened nAb Response

**Figure 6:**
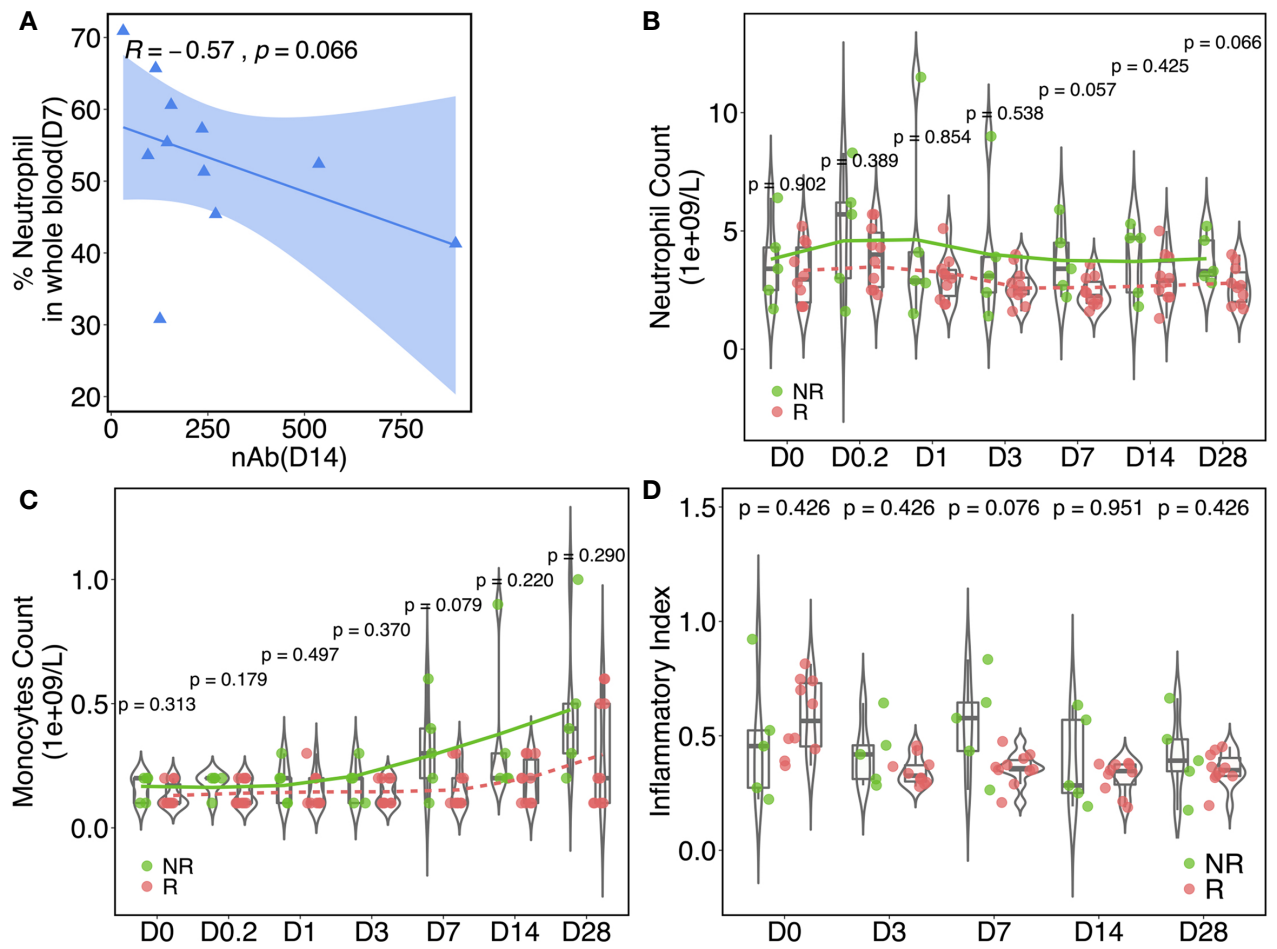
Inflammatory responses are associated with the neutralizing antibody (nAb) response. **(A)** Spearman’s correlation between the nAb titer (day 14) and the neutrophil percentage (day 7) in whole blood. The data plotted are from responders. The *blue shaded area* indicates the confidence interval of the trend line. Kinetics of the percentage of neutrophils **(B)** and monocytes **(C)** in whole blood at baseline and at different time points post-vaccination in responders (Rs) and in non-responders (NRs). **(D)** Inflammation index values in NRs and Rs at different time points pre/post-rTV/HIV-1 (recombinant Tiantan vaccinia-based HIV-1 vaccine) vaccination. The index was calculated according to 27 pre-identified signature genes that are involved in inflammation pathways. For all, *p*-values were calculated using the Wilcoxon test.

Vaccinia inoculation initiated the activation of the AP-1 pathway (51), which is a major pathway mediating the inflammatory response. Our gene profiling data also pointed to a clear association between inflammation and nAb. Neutrophils and monocytes, two immune cell subsets that secrete pro-inflammatory cytokines, can be considered as surrogates of inflammation alternatively. Across all participants, a moderate inverse correlation (*R* = −0.57, *p* = 0.066) was found between the percentage of neutrophils in whole blood on day 7 and anti-vaccinia nAb titer on day 14 (**Figure 6A**). Cell type profiling was assessed in fresh whole blood to calculate the percentages of neutrophils (**Figure 6B**) and monocytes (**Figure 6C**) in circulation. As shown in **Figure 6B**, NRs started with a similar percentage of neutrophils at baseline to Rs, but relatively elevated at hour 4 through day 28 and revealed near significances on days 7 and 28. Moreover, the percentages of monocytes (**Figure 6C**) remained similar in Rs and NRs from baseline through day 3, then followed by a rapid elevation on day 7 in NRs with near significance (*p* = 0.079). We also measured the inflammation index of both groups within the sampling time course. This measurement was calculated based on the expressions of inflammatory pathway-related genes (see *Materials and Methods*). The inflammatory index of the Rs was slightly higher than that of the NRs at baseline, whereas the opposite pattern was observed post-rTV/HIV-1 boost. The highest difference between the NRs and Rs occurred on day 7 (0.551 *vs.* 0.358, *p* = 0.076) (**Figure 6D**).

## Discussion

The different poxvirus vectors that have been explored in HIV vaccine trials elicit distinct immune responses and stimulate different cell signaling pathways (52, 53), highlighting the importance of characterizing the response to each candidate vector. In contrast to the ALVAC canarypox vector used in the RV144 trial, the Tiantan vaccinia virus strain replicates in humans; thus, important differences in the host response to the Tiantan strain might be expected compared to what is known about the ALVAC host response.

Vaccinia virus suppresses innate responses (54) and escapes immune surveillance. PBMCs isolated from smallpox vaccine-recipient NRs (nAb titer < 1:32) (55) have been shown to have significantly lower secretions of IFNα, IFNγ, IL2, and IL4 after *in vitro* vaccinia virus stimulation compared to responders (14). Moreover, the interferon α/β signaling pathway (56) has been shown to be associated with high antibody responders. Consistent with the known role of helper T cells in shaping the humoral immune response (57), we found that the anti-vaccinia nAb titer positively correlated with the vaccinia-specific T-cell response on the population level (**Figure 2C**). However, on both day 14 and 28, some vaccine recipients showed a high nAb titer, yet no T-cell response; conversely, other vaccine recipients showed a high T-cell response, yet no detectable nAb titer. These findings imply a level of independence between the humoral and cellular responses and are also consistent with the observation that vaccine-induced immune responses are often heterogeneous, oftentimes highly so, across individuals (58–61). The limitation of a small sample size, discussed further below, prevented us from further investigating the interplay between the humoral and the cellular response.

Vaccinia infection has been shown to activate the mitogen-activated protein kinase (MAPK)/AP-1 pathway and to inhibit the activation of the pro-inflammatory transcription factor NF-κB (62). Our transcriptional factor network reconstruction showed that AP-1 core genes were present in the networks reconstructed for Rs (**Figure 5A**), but not in those for NRs (**Supplementary Figure S3**). Through further examination of the expressions of AP-1 complex genes, we found that some AP-1-related genes were upregulated at early time points (hour 4 and day 1) in both Rs and NRs (**Figure 5D**). Moreover, NRs had high AP-1 core gene expressions on day 7 (**Figure 5C**). The pro-inflammatory cytokines IL1β, IL6, and TNFα were also highly expressed in NRs, especially on day 7, whereas they were downregulated in most responders on this day. These results suggest that the rTV/HIV-1 booster vaccination induces early activation of the AP-1 genes in individuals who go on to mount a nAb response to the vaccinia virus, but not in individuals who do not. However, on day 3, this pattern switches to the downregulation of the AP-1 genes in Rs, potentially *via* a feedback regulation mechanism. These findings are consistent with proteomic data from a previous study (63), which found that core AP-1 subunits were rapidly upregulated (as early as 2 h) after *in vitro* vaccinia virus infection, with the levels remaining upregulated through 18 h post-infection.

Using PBMCs isolated from Dryvax vaccine recipients (vaccinated between 30 days and 4 years before recruitment) with high *vs.* low anti-vaccinia nAb responses, Kennedy et al. performed an 8-h stimulation with the vaccinia virus and compared the transcriptional profiles of the two groups, reporting that PBMCs from low (*versus* high) responders showed reduced innate antiviral gene expressions (64). A strength of our work compared to the study by Kennedy et al. is that our participants were enrolled in the same trial, with fine kinetic sampling permitting the identification of actual early post-rTV/HIV-1 boost responses. Our data do not answer the question of whether the initiation of the AP-1 pathway per se is essential to generate a nAb response, but they do indicate that the timing of AP-1 activation matters. We hypothesize that, if pro-inflammatory and AP-1 signaling are delayed and do not initiate until day 3 or later, this delayed signaling interferes with the adaptive response. In NRs, many AP-1 core genes were upregulated on day 7, along with IL1β, IL6, and TNFα (**Figure 5C**). Our gene co-expression network and the analysis of various immune responses with nAb titer further indicated that nAb responses were positively correlated with T-cell and B-cell-related modules, but inversely correlated with neutrophil proportions and related pro-inflammatory responses (**Figures 3** and **4**).

A few caveats of this study are that the rTV/HIV-1 construct contains the HIV-1 *gag*, *pol*, and *gp140* genes. Therefore, it is not possible to determine which of the observed responses to the rTV/HIV-1 booster vaccination were due to the vaccinia vector backbone and which were due to the HIV-1 inserts. However, the HIV gene inserts were much smaller compared to the vaccinia genome, suggesting that they may have minimal impact. Secondly, the three HIV-1/DNA primes may have also influenced the vaccine responses. To investigate this possibility, we compared the baseline gene expression profiles in Rs *vs.* NRs and found no significant differences. An additional limitation is the relatively small number of study participants and the relative lack of age/sex diversity, as we could only analyze data from participants in the sub-study who passed the screening criteria and provided informed consent. After smallpox was declared eradicated, routine TVV immunization of the general public in China was stopped. We have not been able to obtain approval from our Ethics Committee to conduct a study on TVV immunization in healthy participants. Thus, studying the responses to TVV in the context of this rTV-based HIV vaccine trial is one of the few viable paths for observing TVV-related genetic and immunological features.

Cumulatively, our results suggest that, after the rTV/HIV-1 booster, early AP-1 activation is required to initiate immune responses. However, resolution of the inflammatory response components by day 3 or day 7 (depending on the component) and the subsequent stimulation of T-cell-mediated immunity are important for developing a nAb response against the vaccinia virus. Neutralizing antibodies may wane with the initial priming, leading to insufficient immunity. These individuals may benefit from additional vaccine doses or adjuvanted versions of the vaccines. A greater understanding of the genetic basis for this insufficient immune response could allow us to tailor vaccination strategies to suit an individual’s needs.

## Data Availability Statement

The datasets presented in this study can be found in online repositories. The names of the repository/repositories and accession number(s) can be found below: https://www.ncbi.nlm.nih.gov/geo/, GSE118976.

## Ethics Statement

The studies involving human participants in this phase 2a trial were reviewed and approved by the Ethics Committee of the Chinese CDC (Ethics Committee project identifier: X111012202). The patients/participants provided written informed consent to participate in this study.

## Author Contributions

JH and SW did the transcriptional experiments. JH did analyses in **Figures 2**–**6** and **Supplementary Figures S1–S3**. DL, MJ, and HP performed the flow cytometry and ELISPOT experiments in **Figure 2**. SW and CL conducted the neutralization assay in **Figure 2**. SW, YL, TZ, and HW organized the clinical study. HW, YH, and YS designed the trial. JH, LC, YH, and YS interpreted the data and wrote the paper. All authors contributed to the article and approved the submitted version.

## Funding

This study was supported by the National S&T Major Project on Major Infectious Diseases (no. 2018ZX10731101), the China–US Cooperation on HIV/AIDS Vaccine Clinical Trial (Chinese Ministry of Science and Technology grant no. 2016YFE0107600), Systems Vaccinology (NSFC-NIH grant 81020108030), a State Key Laboratory for Infectious Disease Prevention and Control (SKLID) Outstanding Youth grant (2019SKLID402), and the National Institute of Allergy and Infectious Diseases of the National Institutes of Health (UM1AI068635 and HVTN SDMC).

## Conflict of Interest

The authors declare that the research was conducted in the absence of any commercial or financial relationships that could be construed as a potential conflict of interest.

## Publisher’s Note

All claims expressed in this article are solely those of the authors and do not necessarily represent those of their affiliated organizations, or those of the publisher, the editors and the reviewers. Any product that may be evaluated in this article, or claim that may be made by its manufacturer, is not guaranteed or endorsed by the publisher.
