# Supplementary material for: Early Pro-Inflammatory Signal and T-Cell Activation Associate With Vaccine-Induced Anti-Vaccinia Protective Neutralizing Antibodies
Source: Front Immunol. 2021 Oct 11;12:737487. doi: 10.3389/fimmu.2021.737487 (PMC8542877; doi:10.3389/fimmu.2021.737487)
Supplement: Supplementary file 1 [file DataSheet_1.docx]

**Supporting information**


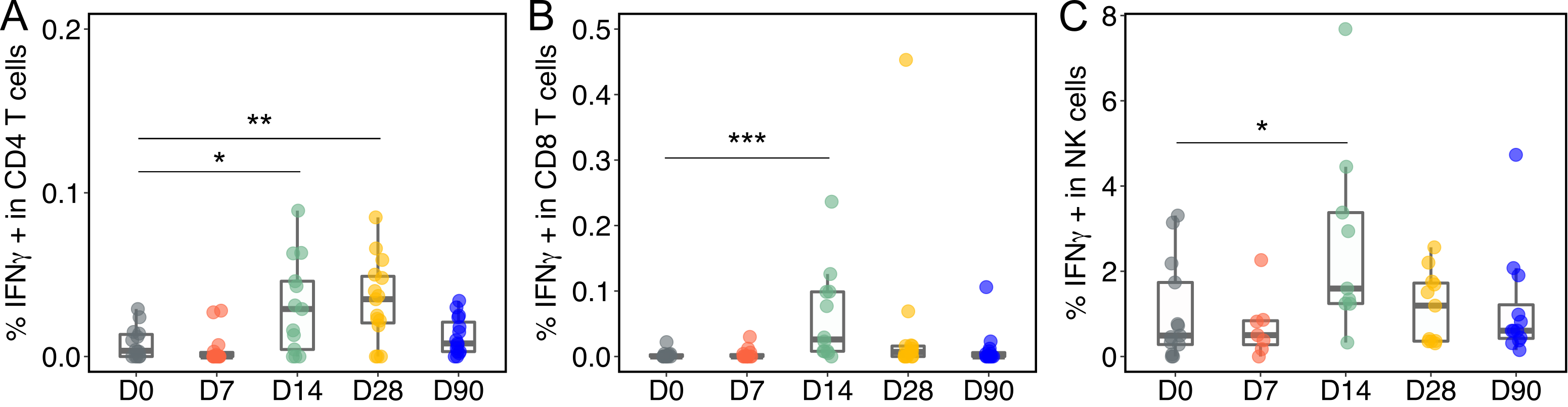


S Fig 1. Kinetics of vaccinia-specific T-cell and NK-cell responses after rTV/HIV-1 boost vaccination.

Vaccinia virus-specific CD4^+^ T-cell (A), CD8^+^ T-cell (B) and NK-cell (C) responses as assessed by intracellular staining assay upon vaccinia antigen specific stimulations. Individual values are presented in box plots. P values of the significance of the difference across two given time points were calculated by the Wilcoxon test.

**
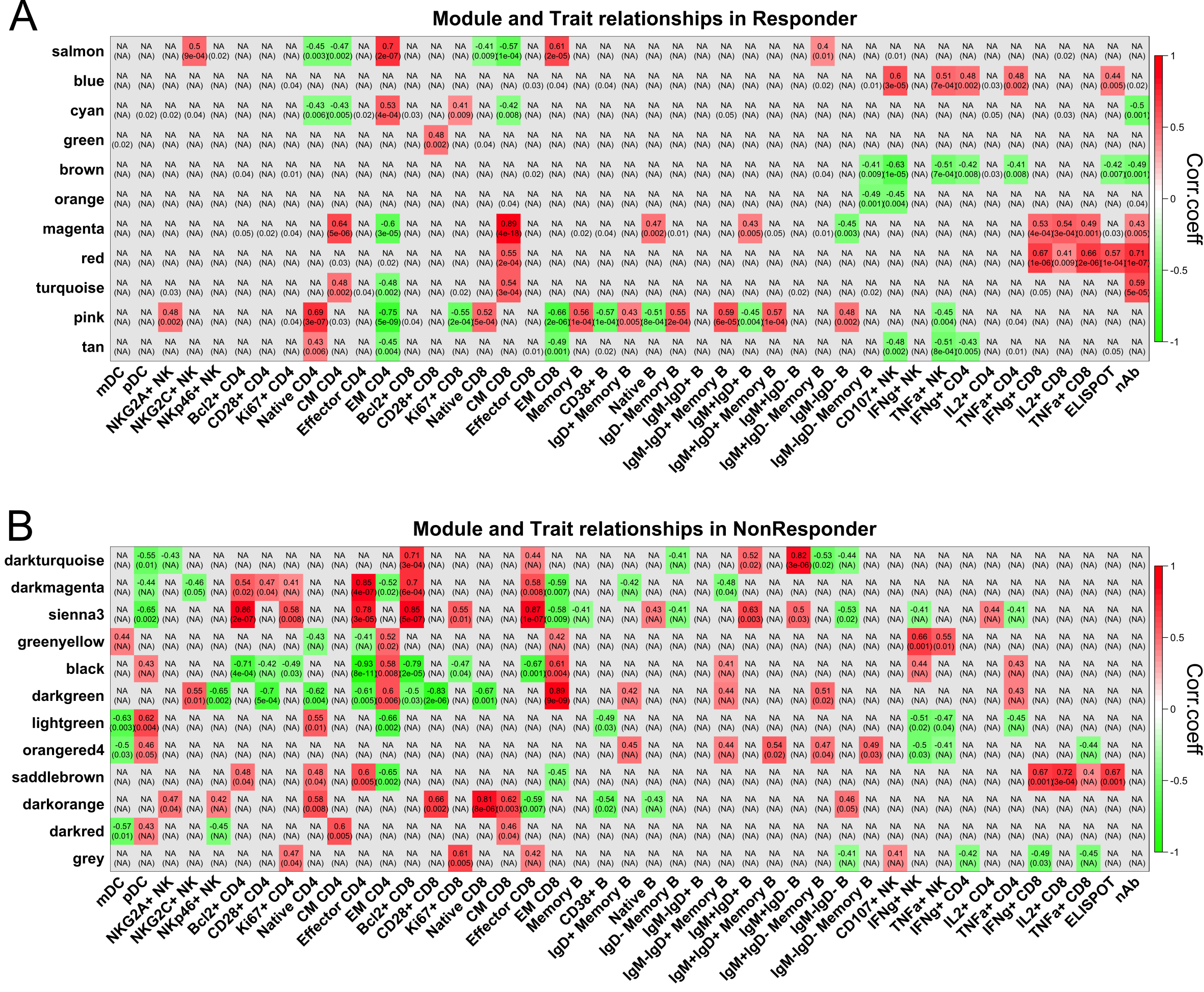
**

S Fig 2. WGCNA co-expression network in Rs and NRs.

Correlations of WGCNA modules and immune response readouts in Rs (A) and NRs (B). Each row in each table corresponds to a module, and each column to an immune response readout. Numbers in the table report the correlations of the corresponding module and immune response readouts. P-values are shown below the correlations in parentheses. The strength and direction of the correlation are indicated by cell shading (red = positive correlation; green = negative correlation).


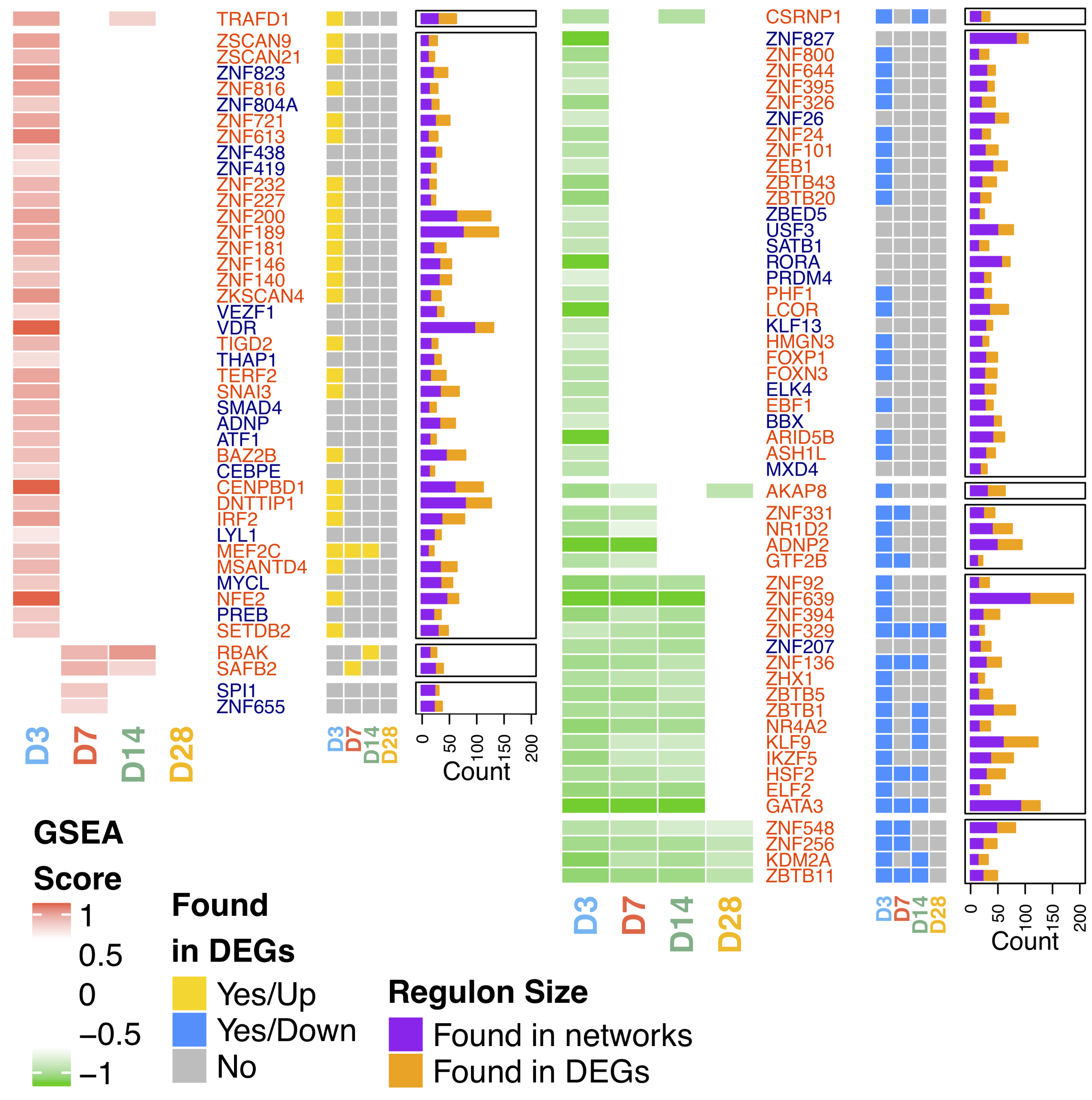


S Fig 3. Reconstruction of transcriptional networks in NRs

Transcriptional networks were reconstructed with expression data in NRs. The identified transcriptional regulatory units were found via network reconstruction. Gene names are shown in red; a yellow square indicates that the DEG was upregulated on the designated time-point, a blue square indicates that the DEG was downregulated on the designated time-point, and a gray square indicates that the DEG was not significantly differentially expressed on the designated time-point. The size of each regulon manipulated by each TF is given in the stacked bar plot. Purple indicates regulons found through the network analysis; mustard indicates regulons present as the DEGs.

S Table 1. Antibodies included in the FACS panels

| Antibody | Catalogue | Company | Assay |
| --- | --- | --- | --- |
| CD123-FITC | 558663 | BD | Monocytes and DC phenotype |
| CD19-PE | 555413 | BD | Monocytes and DC phenotype |
| CD3-ECD | IM2705U | Beckman | Monocytes and DC phenotype |
| CD11c-PECY5 | 551077 | BD | Monocytes and DC phenotype |
| CD56-PECY7 | 335791 | BD | Monocytes and DC phenotype |
| HLADR-APC | 559866 | BD | Monocytes and DC phenotype |
| CD14-A700 | 557923 | BD | Monocytes and DC phenotype |
| CD16-APCH7 | 560195 | BD | Monocytes and DC phenotype |
| CD3-FITC | 555339 | BD | NK phenotype |
| NKG2C-PERCP | FAB138C | RD | NK phenotype |
| NKG2A-PE | FAB1059P | RD | NK phenotype |
| CD20-ECD | IM3607U | Beckman | NK phenotype |
| CD56-PECY7 | 335791 | BD | NK phenotype |
| NKP46-APC | 558051 | BD | NK phenotype |
| CD14-Ax700 | 557923 | BD | NK phenotype |
| CD16-APCH7 | 560195 | BD | NK phenotype |
| IgD-FITC | 555778 | BD | B cell phenotype |
| CD38-PERCP | 551400 | BD | B cell phenotype |
| CD20-ECD | IM3607U | Beckman | B cell phenotype |
| CD27-APCCY7 | 560222 | BD | B cell phenotype |
| IgM-APC | 551062 | BD | B cell phenotype |
| CD3-Ax700 | 557943 | BD | B cell phenotype |
| CD19-PECy7 | 557835 | BD | B cell phenotype |
| TNFα-FITC | 554512 | BD | NK function |
| CD3-PERCP | 347344 | BD | NK function |
| CD14-PE | 555398 | BD | NK function |
| CD19-ECD | A07770 | Beckman | NK function |
| CD56-PECY7 | 335791 | BD | NK function |
| CD107a-APC | 560664 | BD | NK function |
| IFNγ-Ax700 | 557995 | BD | NK function |
| CD16-APCH7 | 560195 | BD | NK function |
| CD154-FITC | 555699 | BD | T cell function |
| CD8-PERCP | 347314 | BD | T cell function |
| IL2-PE | 559334 | BD | T cell function |
| CD3-ECD | IM2705U | Beckman | T cell function |
| CD4-PECY7 | 557852 | BD | T cell function |
| TNFα-APC | 340534 | BD | T cell function |
| IFNγ-Ax700 | 557995 | BD | T cell function |
| CD14-APCH7 | 557831 | BD | T cell function |
